# Low-temperature stress affects reactive oxygen species, osmotic adjustment substances, and antioxidants in rice (*Oryza sativa* L.) at the reproductive stage

**DOI:** 10.1038/s41598-022-10420-8

**Published:** 2022-04-13

**Authors:** Zhenhua Guo, Lijun Cai, Chuanxue Liu, Zhiqiang Chen, Shiwu Guan, Wendong Ma, Guojun Pan

**Affiliations:** 1 Rice Research Institute of Heilongjiang Academy of Agricultural Sciences, Jiamusi, 154026 China; 2 National Engineering Research Center of Plant Space Breeding, South China Agricultural University, Guangzhou, 510642 China; 3 Jiamusi Branch of Heilongjiang Academy of Agricultural Sciences, Jiamusi, 154007 China

**Keywords:** Biochemistry, Plant sciences

## Abstract

The sensitivity of rice to low-temperature stress (LTS), especially at the reproductive stage, is a primary factor of rice yield fluctuation in cold cultivate region. Here, the changes of reactive oxygen species (ROS), osmotic adjustment substances, and antioxidants in different tissues were analyzed during rice growing under low temperatures (LT) at the reproductive stage. Results showed that LTS increases the levels of proline (Pro), soluble protein (SP), glutathione (GSH), superoxidase (SOD), and ascorbate peroxidase (APX) in LJ25 (LTS-resistant) and LJ11 (LTS-sensitive). The activities of catalase (CAT) and peroxidase (POD) were significantly increased in LJ25 but decreased in LJ11 under LTS, while an opposite trend in ROS and malondialdehyde (MDA) was observed in both varieties. Moreover, most physicochemical properties were higher in flag leaves and panicles compared with those in leaf sheaths. The expression patterns of *OsCOIN*, *OsCATC*, *OsMAP1*, *OsPOX1*, and *OsAPX* were the same with phenotypic changes in Pro and the enzymes encoded by them, confirming the accuracy of the physicochemical analysis. Therefore, only CAT and POD increased more in LJ25, suggesting they could be the key factors used for LT-tolerant breeding of rice in cold regions.

## Introduction

Rice (*Oryza sativa* L.) is a thermophilic crop and vulnerable to low-temperature stress (LTS) due to its origin in tropical and subtropical regions^1^. Current unseasonable temperature variations, such as the high frequency of extreme weather, cold damage is a worldwide issue, which is known to reduce rice yield by about 3–5 billion tons annually. Approximately 24 countries located in high-altitude or high-latitude areas are significantly influenced by LTS-induced yield damage^2^. Moreover, the LTS tolerance of indica rice (*O. sativa* L. ssp. *indica*) is much weaker than japonica rice (*O. sativa* L. ssp. *japonica*) due to natural selection based on the contrasting environmental temperatures^3,4^. Due to its sensitivity to LTS, LT threatens rice growth throughout its life span, with the reproductive stage being the most sensitive period^5^. When rice is exposed to LTS at the reproductive stage, LT leads to poor differentiation of branches and spikelets, pollen sterility, reduction in grain number per panicle, and low setting rates, resulting in reduced yield^6^.

The LTS damage to rice is an extremely complicated biophysical and biochemical process. When exposed to LT, rice experiences changes in stability and functioning of the cell membrane, the efficiency of photosynthesis, the amounts of antioxidants and osmoprotectants^7,8^. Therefore, the resistance of rice to LTS is a physicochemical reaction process involving multiple systems, which is influenced by the environmental conditions and the genetic characterizations themselves. LT-tolerant rice shows strong tolerance and adaptation than cold-sensitive ones by adjusting their homeostasis^9^. The antioxidant enzymes, POD, SOD, and CAT, act as a protective enzyme system to limit the levels of free radicals and prevent their damage, maintaining a balance between the antioxidants and free radicals^10^. When exposed to LTS, excessive free radicals, peroxides, and ROS cause severe oxidative damage in rice, whereas the antioxidant defense system diminishes their deleterious impacts on rice^11^. Besides antioxidant enzymes, there are several non-enzymatic antioxidants and osmoprotectants that play important roles in the resistance to various types of abiotic stresses, including drought, heavy metal, and LTS. One of these is GSH, a low-molecular weight reduced sulfur compound^12^. The amino acid proline (Pro) accumulates in response to LTS and has been proposed to enhance tolerance to lts in rice, shown by the balanced relationship between Pro accumulation and stress tolerance^13–15^. Soluble protein (SP) also accumulates in plants under LTS and is also known to act as an osmoprotectant against dehydration damage^16^.

Since rice is a sessile organism exposed to the environment, the whole plant is adversely affected by LTS^17^. The leaf, especially the flag leaf, is the main photosynthetic organ, responsible for the production of energy and organics in rice^18^. LTS is known to induce excessive ROS accumulation^19^, which severely damages membrane lipid composition, chloroplast ultrastructure, the light-harvesting chlorophyll antenna complexes^20^, and the thylakoid structures in rice plants, restricting photosynthesis and reducing energy production^21^. As the major energy sink, the rice panicle is most sensitive to LTS at the reproductive stage^5^. Previous studies have shown that the period of the tetrad to the young microspore stage (YM stage) during the reproductive stage is the most vulnerable to LT^22–24^. Excessive ROS accumulation can destroy the tapetal cells, leading to the degradation of the callose wall and pollen abortion and, ultimately, heavy yield losses^25,26^. The leaf sheath can be either a source or a sink organ during different developmental periods. As a source organ, the leaf sheath plays a vital role in photosynthesis during grain development, which directly affects the plant’s photosynthetic ability during grain filling^27^. The leaf sheath acts as a temporary storage location for photosynthetic products and plays a key role in photosynthetic product transport and grain filling^28^. The leaf sheath also functions as a route for the transportation of photosynthetic products from the leaf to the grain. Therefore, LTS at the reproductive stage can damage the leaf sheath and hinder the physicochemical process, directly affecting the rice yield^29,30^.

Many studies have investigated the regulation of LTS-related genes in rice. Several enzyme-encoding genes are known to be involved in LT tolerance, such as the genes encoding alternative oxidases (*AOX1a*, *AOX1b*)^31^, plastid ω-3 fatty acid desaturase (*OsFAD8*)^32^, an E3 ubiquitin ligase (*OsSRFP1*)^33^, pectin methylesterase 1 (*OsPME1*)^34^, phytase 1 (*OsPHY1*)^35^, and hydroxysteroid dehydrogenase (*OsHSD1*)^36^. The peroxidase gene *OsPOX1* is responsive to various types of abiotic stresses. Northern analysis showed that *OsPOX1* was preferentially expressed in spikelets and was responsive to LTS, while GUS staining showed that *OsPOX1* was expressed in both the aboveground and underground parts of rice during the vegetative stage and in the vasculature and anther at the early flower stage of microspore development^37^. In addition, the cytochrome P450 family member *HAN1* was found to negatively regulate LT tolerance in rice^38^.

Recent studies have reported the mechanism of antioxidant regulation under LTS at the reproductive stage of rice, the majority of studies are concentrated on leaves, and only a few studies have been performed in other tissues, especially in young panicles and leaf sheaths. Therefore, the rice varieties LJ25 (*Oryza sativa* L. ssp. *japonica*) and LJ11 (*Oryza sativ*a L. ssp. *japonica*), which are known to have a significant difference in LT response at the reproductive stage, were selected as the research objects. We studied the response of peroxidase, osmotic regulator, and ROS in the leaves, young panicle, and leaf sheath of the two plants during different stages of LTS in the reproductive stage, providing a theoretical basis for high yield and high-quality production of LT tolerant rice breeding.

## Materials and methods

### Materials

We chose LJ25 and LJ11, the two conventional *Japonica* rice cultivars cultivated by the Rice Research Institute of Heilongjiang Academy of Agricultural Sciences and planted in cold regions, as the experimental materials in this study. LJ11 is a cultivar with a high yield but low LTS tolerance at the reproductive stage, which is planted in the third accumulative temperature zone of Heilongjiang, with the effective accumulative temperature (≥ 10 °C is about 2400 °C ) for maturity. The seed setting rate of LJ11 has been reported to be only 6.18% after eight days of 15 °C LTS treatment^25^. LJ25 is another high-yield and good-quality cultivar planted in the same temperature zone in the Heilongjiang Province as LJ11. Compared with LJ11, LJ25 is more tolerant to LTS at the reproductive stage, whose seed setting rate was approximately 91.9–93.3% treated by 18 ± 0.2 °C cold water for 10 days in 2007 and 2008^39^.

### Growing condition

The pot experiment was performed in the artificial climate room of Rice Research Institute of Heilongjiang Academy of Agricultural Sciences (Jiamusi, Heilongjiang, China) in 2019. The cultivation of the experimental materials was according to the methods of Guo et al.^39^ with more details. The diameter of the pot was 25 cm, and the height was 23 cm. Before the experiment, each pot was filled with 8 kg of dry paddy soil from the paddy fields of the experimental farm of Rice Research Institute of Heilongjiang Academy of Agricultural Sciences. The basic physical and chemical properties of soil were pH 6.22, organic matter 36.56 g/kg, alkali hydrolysis N, available P, and available K were 105.35 mg/kg, 82.56 mg/kg, and 92.6 mg/kg, respectively. After disinfection, seed soaking, and germination, the seeds of LJ25 and LJ11 were seeded on a seedling plate containing the paddy soil and raised in a plastic shed on April 16. On May 16, when the rice seedlings grew to 3 leaves, and 1 heart stage, the seedlings with the same development process were selected and transplanted into plastic pots. Twenty seedlings with consistent growth were selected and evenly planted in each pot, and each cultivar was planted in twelve pots. Extra tillers of each plant were removed to ensure the consistent growth process of each plant, leaving only the main stem and grown at normal growing condition (28 °C day/22 °C night, 12 h-light/12 h-dark photoperiod, 80% RH). The amounts of fertilization were the same as that of field fertilizer levels in the experimental farm of Rice Research Institute of Heilongjiang Academy of Agricultural Sciences (nitrogen 100 kg/hm^2^, phosphorus 80 kg/hm^2^, and potassium 80 kg/hm^2^).

### LT treatment and sample preparation

Here, the LT treatment and sample preparation were performed following the methods of the cool air treatment indoors^39^. The meiosis at the reproductive stage is known to be the most sensitive stage to LTS^40^. However, the determination of the pollen development period through microscopic examination was time-consuming and laborious, although precise. Therefore, the pollen development period was usually estimated based on the auricle distance method. Briefly, the flag leaf’s auricle was approximately 5 cm beneath the of the penultimate leaf’s auricle^40,41^. Till this stage, half of both the cultivars were transferred to another artificial climate room maintained at 12 °C for 4 days (12 h-light/12 h-dark photoperiod, 80% RH) and then returned to the original room till maturity. Next, 0.5 g of fresh flag leaf and 0.5 g of leaf sheath were collected from each group after 0 days, 2 days, and 4 days of treatment at 12 °C, respectively. While, 0.5 g of fresh young spikelets, about 3.5–4.5 mm length, were plucked and collected from the upper third of the panicles after 0 days, 2 days, and 4 days of 12 °C treatment. All the samples of fresh flag leaf, leaf sheath, and fresh young spikelets were immediately frozen in liquid N_2_ and stored at − 80 °C. LTS resistance was evaluated on seed setting rates (SSRs) of the main spikelet.

### Determination of the content of MDA, Superoxide (O_2_-), and hydrogen peroxide (H_2_O_2_)

The MDA content was determined following the method of Guo et al.^41^. The absorbance of MDA was measured at 440, 532, and 600 nm on UV–Vis Spectrophotometer (Mettler-Toledo UV5Bio, Switzerland). The superoxide (O_2_-) content was determined following the method of Batool et al.^42^. The phosphate buffer (pH 7.8, 0.5 mL), p-aminobenzene sulfonic acid (17 mM, 1 mL), hydroxylammonium chloride (1 mM, 1 mL), and α-naphthylamine (7 mM, 1.0 mL) were mixed, incubated at 25 °C for 60 min and then measured at 530 nm for absorbance. The H_2_O_2_ content was measured following the method of Song et al.^19^ with minor modifications. Each sample was ground into a homogenate with trichloroacetic acid (0.1%) and then centrifuged at 10,000×*g* at 4 °C for 20 min. Next, 1 ml of the supernatant was mixed with 2 mL of KI (1 M) and 1 mL of K_2_PO_4_ buffer, and the absorbance was measured at 390 nm after 1 h darkness treatment.

### Determination of antioxidant enzymes activities

Frozen samples of the flag leaves, leaf sheath, and young spikelets were mixed with potassium phosphate buffer (50 mM, pH 7.8), containing 1% polyvinylpyrrolidone, respectively, and then ground with a pre-cooled pestle and mortar. The prepared crude enzyme extract was incubated with the supernatant after homogenization and centrifuged at 15,000×*g* at 4 °C for 20 min. The activities of POD (EC 1.11.1.7), SOD (EC 1.15.1.1), and CAT (EC 1.11.1.6) were measured following the method of Guo et al.^39^. The activity of APX (EC 1.11.1.11) was measured following the method of Sato et al.^41^.

### Determination of the contents of osmotic adjustment substances

Free proline was estimated following the method of Bates et al. with minor modifications^43^. Samples were submerged in 5 mL of sulphosalicylic acid (3%, 100 °C for 10 min); 2 mL of the extract was mixed with ninhydrin reagent containing glacial acetic acid and then incubated for 30 min at 100 °C. After cooling in ice water, 4 mL of toluene was added to the mixture and then measured at 520 nm to determine the proline content. The SP content was estimated via the BCA method following the protocol of Campion et al.^44^. The GSH content was measured according to the method described by Gautam et al.^45^.

### Determination of the expression levels of the related genes

*OsCOIN*, *OsCATC*, *OsMAP1*, *OsAPXa*, and *OsPOX1* were selected for determining the expression of different tissues in LJ25 and LJ11 under LTS. Total RNA in all samples was extracted using the respective RNA mini extraction kit (Invitrogen), following the manufacturer’s instructions. Primer 3 software was used for designing the primers, The Supplementary Table S1 shows the list of primers. QuantiNova™SYBR®Green PCR kit (Qiagen Inc., Duesseldorf, Germany) was used to perform the qRT-PCR reactions, which was carried out on an ABI StepOne Plus system. The 2^−ΔΔCT^ method was used for qRT-PCR data analysis with three replicates in each reaction. Actin1 was selected as the reference gene.

### Statistical analyses

All the data obtained were statistically analyzed using the SPSS 19.0 (v20.0, SPSS Inc., Chicago, USA) for variance (ANOVA) analysis. The least significant difference (LSD) test was used to determine the significant differences among treatments (*P* < 0.05).

### Ethics approval and consent to participate

The seeds were kindly provided by the Rice Research Institute of Heilongjiang Academy of Agricultural Sciences, Jiamusi, China. In this study, the experimental research and field studies on plants, including collection of plant material, complied with relevant institutional, national, and international guidelines and legislation.

## Results

### Effects of LTS on seed setting rates at the reproductive stage

**Figure 1:**
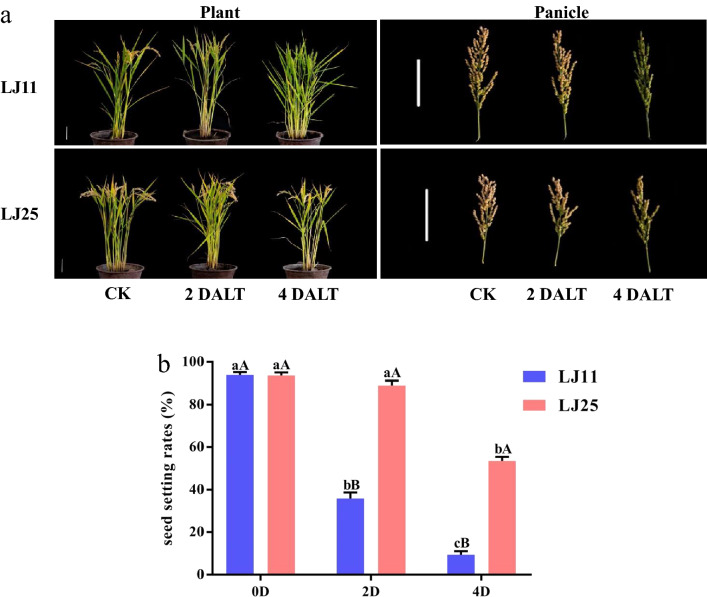
Phenotypes and seed setting rates of LJ11 and LJ25 under LTS at the reproductive stage. (**a**) The plant types (scale bar: 10 cm) and panicles (scale bar: 10 cm) of LJ11 and LJ25. ‘CK’ represents ‘control group.’ ‘2 DALT’ and ‘4 DALT’ represent after 2 days and 4 days of LTS treatment, respectively. (**b**) Seed setting rates of LJ11 and LJ25.‘CK’ represents ‘control group.’ ‘2D’ and ‘4D’ represent after 2 days and 4 days of LTS treatment, respectively. Lower-case letters represent significant differences among the three treatments for each genotype, upper-case letters represent significant differences between the two genotypes at each treatment.

In the present study, we analyzed the SSRs of LJ11 and LJ25 during the LTS after 0 day, 2 days, and 4 days. As shown in Fig. 1, the SSR of LJ11 and LJ25 was 93.3% and 93.7% under normal growing conditions (CK group), respectively, without significant difference between the two cultivars. While, the SSRs in LJ11 significantly decreased to 37.90 % and 8.97% at the 2 and 4 days after LTS (2 DALT and 4 DALT), respectively, suggesting that the spikelets were almost entirely sterile at the end of the LTS. In LJ25, the SSR was only significantly decreased at 4 DALT (59.77%). Moreover, the SSRs in LJ25 after 2 days and 4 days of LTS were both significantly higher than those in LJ11 (Fig. 1 & Table S2).

### Response of MDA, O_2_- and H_2_O_2_ to LTS at the reproductive stage

**Figure 2:**
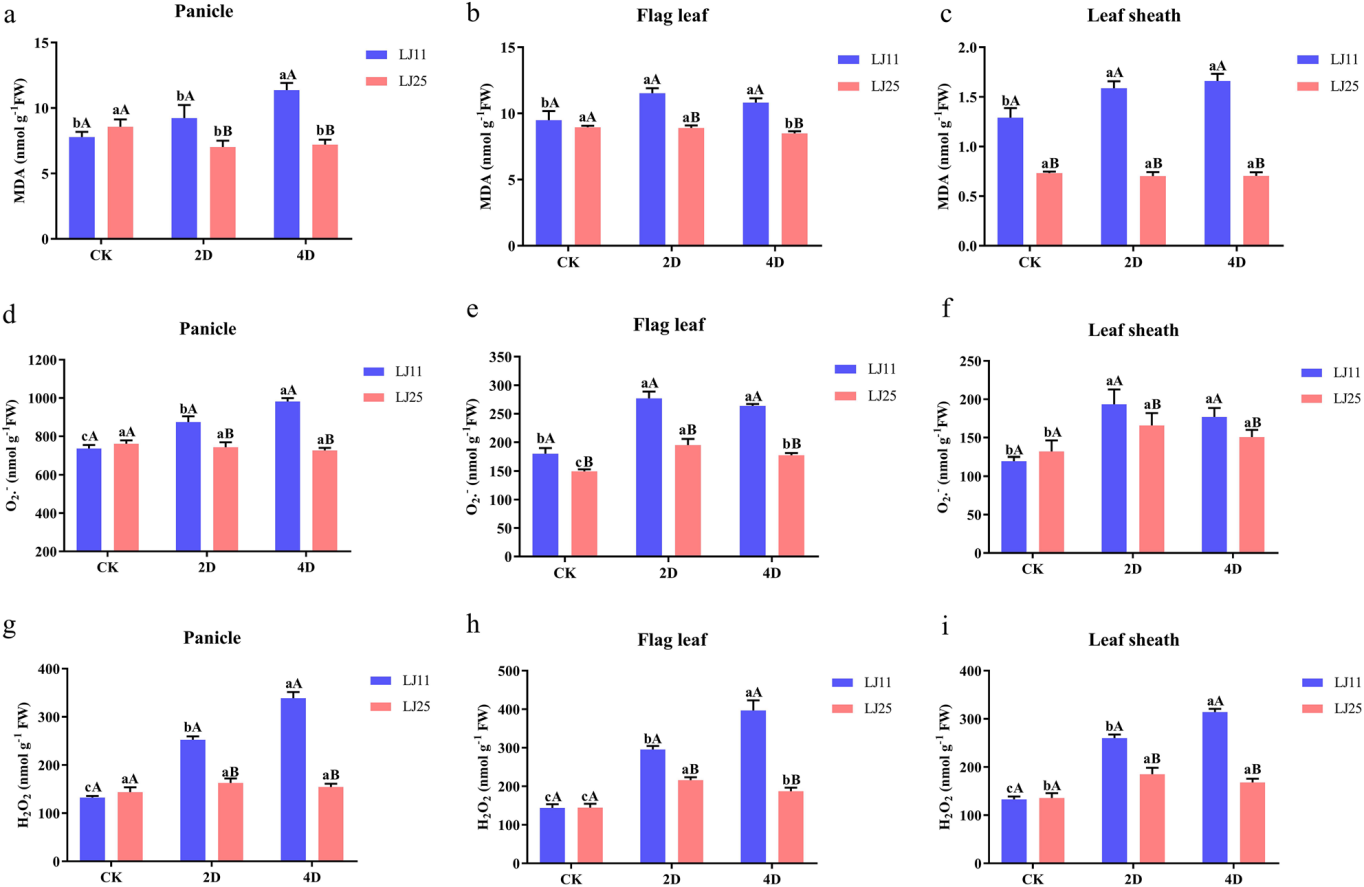
MDA, O_2_-, and H_2_O_2_ levels in different tissues of LJ25 and LJ11 in response to LTS treatment at the reproductive stage. (**a**–**c**) Changes in MDA levels in the panicle, flag leaf, and leaf sheath of LJ11 and LJ25, respectively. (**d**–**f**) Changes in O_2_- levels in the panicle, flag leaf, and leaf sheath of LJ11 and LJ25, respectively. (**g**–**i**) Changes in H_2_O_2_ levels in the panicle, flag leaf, and leaf sheath of LJ11 and LJ25, respectively. ‘CK’ represents ‘control group.’ ‘2D’ and ‘4D’ represent after 2 days and 4 days of LTS treatment, respectively. Lower-case letters represent significant differences among the three treatments for each genotype; upper-case letters represent significant differences between the two genotypes for each treatment. Data are the means and standard errors of three replicates (n = 3). Data with different letters indicate statistically significant differences among the treatments according to Duncan’s multiple range test (*P* < 0.05).

MDA was reported to accumulate under LTS and investigated here. As shown in Fig. 2a–c, the MDA. The MDA contents of LJ11 at 2 DALT increased by 9.24%, 21.46% (*P* < 0.05), and 22.91% (*P* < 0.05) in panicle, flag leaf, and leaf sheath, respectively, compared to the control group (Fig. 2a–c & Table S3). At 4 DALT, the MDA levels increased significantly by 46.12%, 13.96%, and 28.67% in panicle, flag leaf, and leaf sheath, respectively. While in LJ25, the MDA decreased by 18.07% (*P* < 0.05), 0.62%, and 4.15% at 2 DALT in panicle, flag leaf, and leaf sheath, respectively, compared to the control group. Moreover, the MDA levels decreased by 15.83% (*P* < 0.05), 5.81% (*P* < 0.05), and 4.15% at 4 DALT in panicle, flag leaf, and leaf sheath, respectively. As an indicator of membrane lipid peroxidation, the increase or decrease in MDA levels reflected the degree of cell membrane lipid peroxidation and resistance of plants to stress conditions.

The O_2_- content in the panicle of LJ11 significantly increased at 2 and 4 DALT (18.84% and 33.49%, respectively), whereas decreased slightly at 2 and 4 DALT (2.43% and 34.60%, respectively) in LJ25. The response of O_2_^-^ in flag leaf and leaf sheath were both significantly increased at 2 and 4 DALT, in both LJ11 and LJ25 (Fig. 2d–f & Table S3). Moreover, both the MDA and the O_2_^-^ levels were significantly higher in LJ11 than in LJ25 at 2 and 4 DALT.

The H_2_O_2_ levels in the panicle of LJ11 significantly increased by 90.67% and 155.88% at 2 and 4 DALT, respectively, which was 13.42% and 7.40% at 2 and 4 DALT in LJ25 (not significant). In the flag leaf, the H_2_O_2_ levels exhibited the same trends as those in the panicle (105.14% and 175.39% increased at 2 and 4 DALT, respectively), while it was significantly increased by 49.03% and 29.16% at 2 and 4 DALT in LJ25. In the leaf sheath, the response of H_2_O_2_ to LTS showed the same pattern as flag leaf, with 96.10% and 136.70% in LJ11, and 36.46% and 24.05% in LJ25 increasing at 2 and 4 DALT, respectively (Fig. 2g–i & Table S3).

### Response of antioxidant enzymes to LTS at the reproductive stage

**Figure 3:**
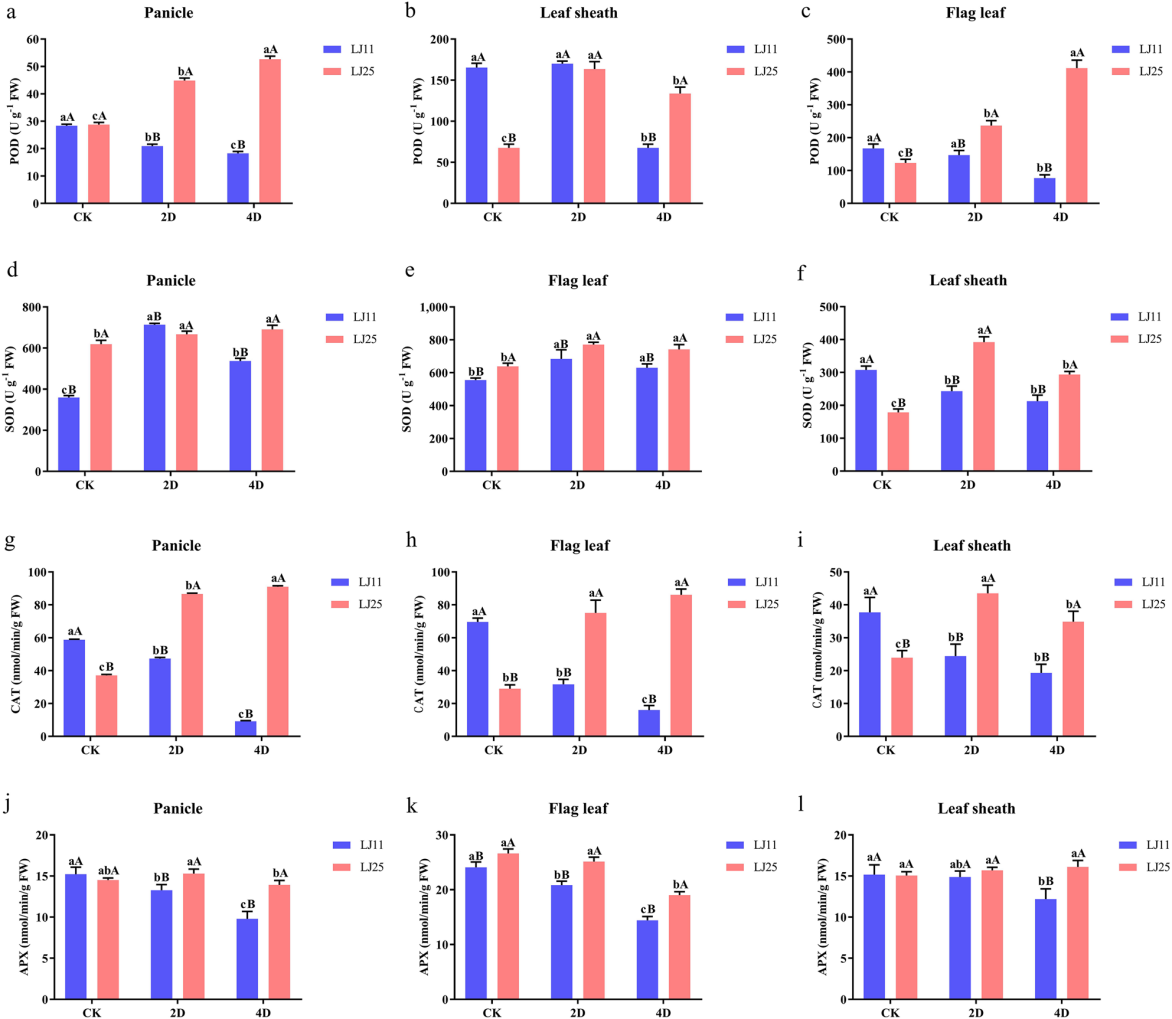
Responses of antioxidant enzymes to LTS in different tissues of LJ25 and LJ11 at the reproductive stage. (**a**–**c**) Changes in POD activities in the panicle, flag leaf, and leaf sheath of LJ11 and LJ25, respectively. (**d**–**f**) Changes in SOD activities in the panicle, flag leaf, and leaf sheath of LJ11 and LJ25, respectively. (**g**–**i**) Changes in CAT activities in the panicle, flag leaf, and leaf sheath of LJ11 and LJ25, respectively. (**j**–**l**) Changes in APX activities in the panicle, flag leaf, and leaf sheath of LJ11 and LJ25, respectively. ‘CK’ represents ‘control group.’ ‘2D’ and ‘4D’ represent after 2 days and 4 days of LTS treatment, respectively. Lower-case letters represent significant differences among the three treatments for each genotype; upper-case letters represent significant differences between the two genotypes for each treatment. Data are the means and standard errors of three replicates (n = 3). Data with different letters indicate statistically significant differences among the treatments according to Duncan’s multiple range test (*P* < 0.05).

The activity of POD in the panicle of LJ25 increased significantly increased at 2 and 4 DALT (55.97% and 82.96%, respectively), while decreasing at 2 and 4 DALT by 26.36% and 35.64% in LJ11, respectively. In the flag leaf, the activity increased significantly by 92.15% and 234.19% at 2 and 4 DALT in LJ25, while decreasing significantly by 12.26% and 54.07% in LJ11, respectively. However, in the leaf sheath of LJ11, the activity of POD decreased significantly only at 4 DALT (59.14%). In addition, except for the control group in the panicle and the activity at 2 DALT in flag leaf, the activity of POD in LJ25 were all significantly higher than that at the same DALT in LJ11 (Fig. 3a–c & Table S4).

The levels of SOD increased significantly by 98.30% and 49.18% at 2 and 4 DALT in LJ11, which were 7.67% and 11.53% in LJ25, respectively. While similar changes occurred in flag leaf between the two cultivars, which were 23.32% and 13.63% in LJ11, and 20.74% and 16.18% in LJ25, respectively. However, in the leaf sheath, the activity of SOD decreased significantly by 20.93% and 30.79% at 2 and 4 DALT in LJ11, while increased significantly by 119.52% and 64.52% in LJ25, respectively. In addition, the activity of SOD was significantly higher in the control group of leaf sheath and 2 DALT of panicle in LJ11 than that in LJ25 (Fig. 3d–f & Table S4).

The activity of CAT in LJ11 decreased significantly at 2 and 4 DALT by 19.30% and 84.32%, 54.51% and 76.96%, 35.25% and 48.87% in the panicle, flag leaf, and leaf sheath, respectively (Fig. 3g–i & Table S4); while in contrast, it was 133.33% and 145.40%, 158.37% and 196.27%, 81.74% and 45.60% in the panicle, flag leaf and leaf sheath of LJ25, respectively. Moreover, except for the control groups of all the tissues, the activities of CAT were remarkably higher in LJ25 than that in LJ11.

The activities of APX in panicle were significantly repressed by 12.76% and 35.71% at 2 and 4 DALT in LJ11 while decreasing significantly by 8.88% at 4 DALT compared with that at 2 DALT in LJ25 (Fig. 3j–l & Table S4). In the flag leaf, the APX significantly decreased in LJ11 by 13.46% and 40.18% at 2 and 4 DALT, respectively, while decreased significantly at 4 DALT in LJ25 by 28.55%. No remarkable changes of the APX occurred in the leaf sheath of LJ25 while decreased significantly at 4 DALT in LJ11 by 19.64%.

### Response of several osmotic adjustment substances to LTS at the reproductive stage

**Figure 4:**
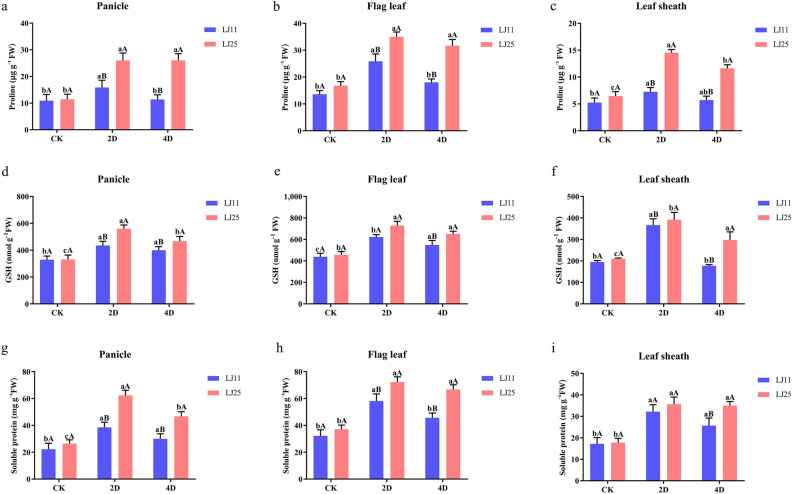
Levels of Pro, GSH, and SP in response to LTS at the reproductive stage in different tissues of LJ25 and LJ11. (**a**–**c**) Changes in Pro levels in the panicle, flag leaf, and leaf sheath of LJ11 and LJ25, respectively. (**d**–**f**) Changes in GSH levels in the panicle, flag leaf, and leaf sheath of LJ11 and LJ25, respectively. (**g**–**i**) Changes in SP levels in the panicle, flag leaf, and leaf sheath of LJ11 and LJ25, respectively. ‘CK’ represents ‘control group.’ ‘2D’ and ‘4D’ represent after 2 days and 4 days of treatment, respectively. Lower-case letters represent significant differences among the three treatments for each genotype; upper-letters represent significant differences between the two genotypes for each treatment. Data are the means and standard errors of three replicates (n = 3). Data with different letters indicate statistically significant differences among the treatments according to Duncan’s multiple range test (*P* < 0.05).

As a non-enzymatic antioxidant, the content of Pro in LJ11 only increased at 2 DALT in the panicle, flag leaf, and leaf sheath by 45.32%, 90.43%, and 38.36%, respectively; however, Pro levels increased significantly by 127.15% and 127.31%, 108.59% and 88.83%, 125.53% and 80.41% at 2 and 4 DALT in panicle, flag leaf, and leaf sheath of LJ25, respectively (Fig. 4a–c & Table S5).

The GSH in different tissues of both LJ11 and LJ25 was significantly increased during the cold stress treatment, except at DALT 4 in the leaf sheath of LJ11. Thus, 32.31% and 21.41%, 42.16% and 25.25%, 87.80% and − 9.09%, were increased at 2 and 4 DALT in the panicle, flag leaf, and leaf sheath of LJ11, respectively, while those were 69.03%, 41.04% and 59.75% and 42.67%, 86.96% and 41.69% in the same tissues of LJ25 (Fig. 4d–f & Table S5).

SP is another non-enzymatic antioxidant, which increased significantly by 73.18%, 80.46%, 86.67% and 34.89%, 41.65%, and 48.88% at 2 and 4 DALT in the panicle, flag leaf, and leaf sheath of LJ11, respectively, while its levels were 135.70%, 94.90%, 100.60% and 76.53%, 79.68% and 96.96% in LJ25, respectively (Fig. 4g–i & Table S5).

### Responses of several antioxidant enzymes and non-enzymatic antioxidant-related genes to LTS at the reproductive stage

**Figure 5:**
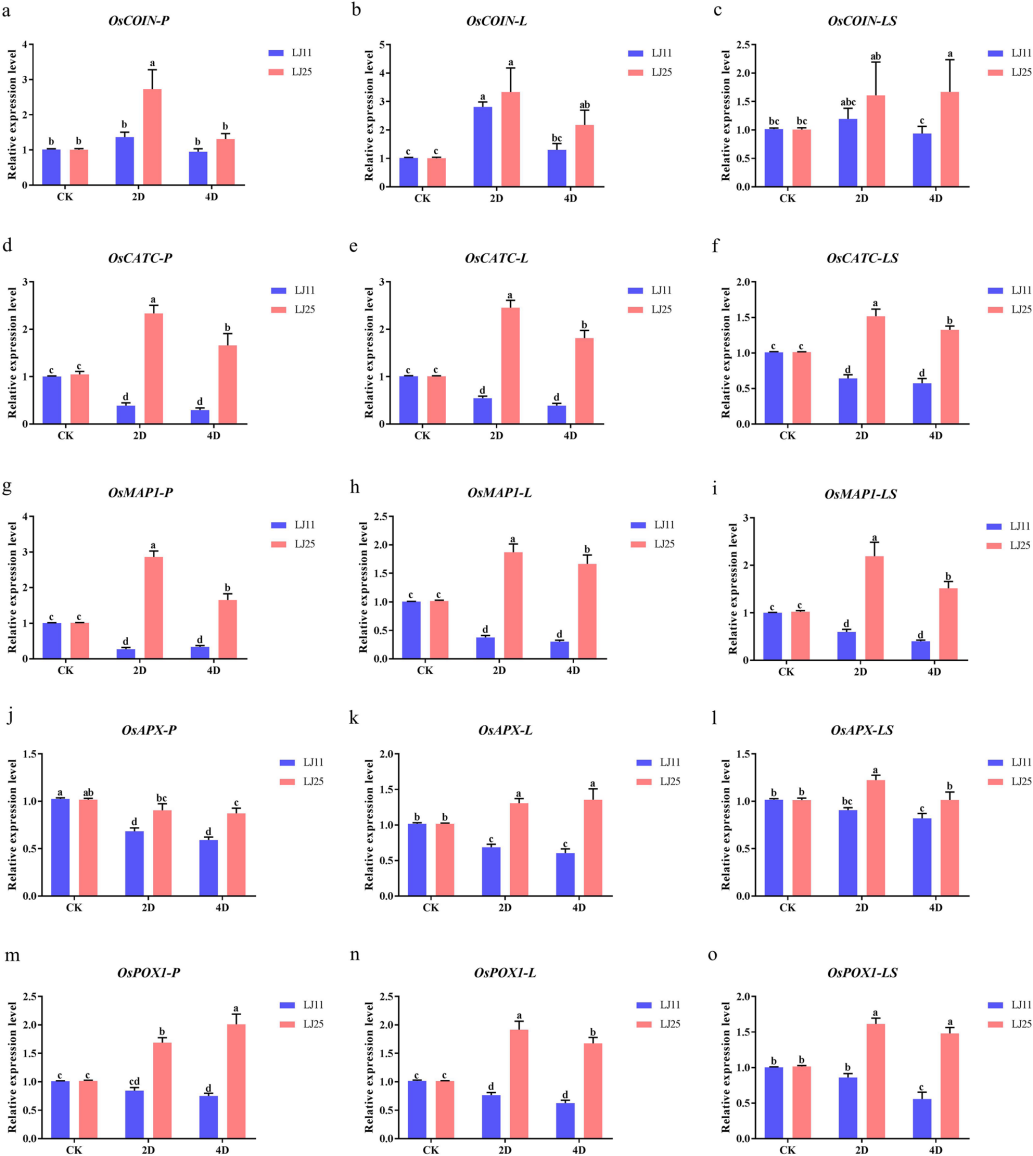
Changes in gene expression in response to LTS at the reproductive stage in different tissues of LJ25 and LJ11. (**a**–**c**) Changes in *OsCOIN* expression in the panicle, flag leaf, and leaf sheath of LJ11 and LJ25, respectively. (**d**–**f**) Changes in *OsCATC* expression in the panicle, flag leaf, and leaf sheath of LJ11 and LJ25, respectively. (**g**–**i**) Changes in *OsMAP1* expression in the panicle, flag leaf, and leaf sheath of LJ11 and LJ25, respectively. (**j**–**l**) Changes in *OsAPX* expression in the panicle, flag leaf, and leaf sheath of LJ11 and LJ25, respectively. (**m**–**o**) Changes in *OsPOX1* expression in the panicle, flag leaf, and leaf sheath of LJ11 and LJ25, respectively. ‘CK’ represents ‘control group.’ ‘2D’ and ‘4D’ represent after 2 days and 4 days of LTS treatment, respectively. Lower-case letters represent significant differences among the three treatments for each genotype. ‘CK’ represents ‘control group.’ ‘2D’ and ‘4D’ represent after 2 days and 4 days of LTS treatment, respectively. Lower-case letters represent significant differences among the three treatments for each genotype; upper-case letters represent significant differences between the two genotypes for each treatment. Data are the means and standard errors of three replicates (n = 3). Data with different letters indicate statistically significant differences among the treatments according to Duncan’s multiple range test (*P* < 0.05).

Under LTS, the changes in physiological and biochemical indexes were accompanied by the changes in their expressions of regulatory genes. Here, we analyzed the expression of *OsCOIN*, *OsCATC*, *OsMAP1*, *OsAPXa*, and *OsPOX1*, related to antioxidase and non-enzymatic antioxidant encoded genes, from different tissues of the two cultivars. The expression of *OsCOIN* in LJ11 increased by 34.24 and − 6.45%, 176.52 and 28.24%, and 17.64 and − 7.57% at 2 and 4 DALT in the panicle, flag leaf, and leaf sheath, respectively, compared to the control groups, while those in LJ25 were 170.87 and 30.25%, 230.89 and 116.22%, and 59.67 and 65.72%, respectively (Fig. 5a–c & Table S5). The expression of *OsCATC* decreased by 61.37 and 70.56%, 45.86 and 61.42%, and 36.41 and 43.12% at 2 and 4 DALT in the panicle, flag leaf, and leaf sheath of LJ11, respectively, compared to the control groups, while those in LJ25 were increased by 123.00 and 58.45%, 143.42 and 79.82%, and 49.90 and 30.93%, respectively (Fig. 5d–f & Table S5). The expression of *OsMAP1* was downregulated by 72.93 and 66.58%, 62.37 and 69.83%, and 40.38 and 60.11% at 2 and 4 DALT in the panicle, flag leaf, and leaf sheath of LJ11, respectively, compared to the control groups, while in LJ25 they were upregulated by 182.10 and 63.58%, 83.83 and 63.70%, and 113.36 and 47.38% in the panicle, flag leaf and leaf sheath, respectively (Fig. 5g–i & Table S5). The expression of *OsAPXa* was downregulated by 33.31 and 42.25%, 32.43 and 40.56%, and 10.84 and 19.32% at 2 and 4 DALT in the panicle, flag leaf, and leaf sheath of LJ11, respectively, related to the control groups, while in LJ25, it was downregulated by 11.16 and 14.33% at 2 and 4 DALT in the panicle, and upregulated by 28.40 and 33.20%, and 20.82 and 0.16% in the flag leaf and leaf sheath, respectively (Fig. 5j–l & Table S5). The expression of *OsPOX1* in LJ11 was downregulated by 16.41 and 25.84%, 24.54 and 38.45%, and 14.21 and 44.33% at 2 and 4 DALT in the panicle, flag leaf, and leaf sheath, respectively, related to the control groups, while those in LJ25 were upregulated by 66.33 and 98.12%, 89.25 and 65.51%, and 59.01 and 45.80%, respectively (Fig. 5m–o & Table S5).

## Discussion

Since rice is grown on the ground and immovable, the type of environmental conditions is crucial for rice survival and development, especially the unfavorable ones, of which LTS is a principal element. As the largest province of rice in north China, Heilongjiang is also an important base of rice commodity grain in China (70% of rice production as a commodity, www.zzys.moa.gov.cn). However, since it is located in the northernmost region of China, LTS, especially on the reproductive stage, seriously restricts the safe production of rice. Thus, LJ25 and LJ11 were selected for further comprehensive investigation into the metabolic regulatory mechanisms underlying LT tolerance in rice exposed to LTS at the reproductive stage. LJ25 and LJ11 present significantly different LT tolerance at the reproductive stage. LJ25 is one of the strongest LTS resistant crops and is widely cultivated in the third accumulative temperate zone in Heilongjiang Province^5^, representing a strong LT tolerance of rice in the cold region at the reproductive stage. LJ11 with good agronomic and yield characters is weakly resistant to LTS when cultivated in the same temperate region^5^. In addition, LJ25 and LJ11 underwent simultaneous meiosis, ensuring the accuracy of the LTS treatment period and the outcomes.

During its evolution, rice has been exposed to a variety of biotic and abiotic stresses and has thus acquired specific physiological adaptations for survival in harsh environments. Antioxidant defense systems play key roles in the alleviation of oxidative damage induced by LTS and include the modulation of osmotic conditions and the coordination of enzymatic and non-enzymatic antioxidants^7,46,47^. Rice produces substantial amounts of ROS under LTS conditions, leading to ROS imbalances that affect the cell’s metabolism and damage intracellular proteins, membrane lipids, and DNA^48^. As the first line of defense, SOD is known to detoxify O_2_^-^ to form H_2_O_2_ and O_2_, while POD, CAT, and APX are the key enzymes that convert H_2_O_2_ into H_2_O^49^. In this study, along with the significant increase in the amount of O_2_- observed in all the investigated tissues of LJ11 and LJ25, it was found that SOD activities were also significantly increased, except in the leaf sheath of LJ11. However, as the next step, the activities of POD, CAT, and APX and the concentrations of H_2_O_2_ and MDA responded differently in the various samples. The levels of MDA and H_2_O_2_ in LJ11 were all significantly increased under LTS while only slightly or even decreasing in LJ25. The POD and CAT levels decreased significantly in LJ11 while increasing remarkably in LJ25. However, the activities of APX in LJ11 and LJ25 showed the same trend, namely, downregulation in all samples in response to LTS. CAT is mainly known to scavenge and break down the H_2_O_2_ produced via the fatty acid oxidation photorespiration reaction, reducing the peroxidation of membrane lipids^50^, and POD is known to decompose H_2_O_2_ using phenol as the substrate^51^. When exposed to continuous LTS, the cytosolic concentrations of the osmotica (SP and Pro) increased, reducing the water potential and alleviating cellular injury. Proline is known to be positively related to plants' responses to LTS and can be used as an indicator to reflect the LT tolerance of plants and the degree of LTS suffered by plants^52^. In this study, both SP and Pro were found to be significantly increased under LTS in the two cultivars, consistent with the previous reports^53,54^, suggesting that they played positive roles in the physiological response to LTS at the reproductive stage of rice in cold regions. The AsA-GSH cycle, formed by GSH and AsA, is known to be an important route for ROS scavenging, where GSH and AsA reduce ROS directly or act as an enzyme–substrate system to remove ROS^55^. The levels of GSH in both LJ25 and LJ11 were significantly increased after LTS treatments, showing that GSH was also a positive regulator of LTS in rice in cold regions. Moreover, the POD and CAT were significantly upregulated in LJ25 and downregulated in LJ11, suggesting that they played key roles in the response of rice to LTS in cold regions.

Since plants are continuously exposed to the environment, all the tissues are threatened by LTS. The flag leaves are the most important source organs in functional leaves and play a vital role in grain yield^56^. As the main energy storage organs, exposure of rice panicle to LT at the reproductive stage directly causes male sterility and seriously affects rice yield^57^. Leaf-sheaths have different physiological functions in different growth stages of rice, where they could act as flow organs or source and sink organs^58^. Here we investigated the responses of the panicle, flag leaf, and leaf sheath on LJ25 and LJ11 to LTS at the reproductive stage. Previous studies have reported that the antioxidant enzymes of flag leaf increase along with the ROS accumulation to maintain the free radicals at appropriate levels^59,60^. Herein, the amounts of O_2_-, H_2_O_2_, MDA, GSH, APX, Pro, and SP in the panicle, flag leaves, and leaf sheath showed the same changing trends in both LJ11 and LJ25, which were approximately consistent with previous reports. The activities of POD and CAT in all the investigated tissues of LJ11 decreased steadily and significantly with LTS, while significantly increased in LJ25. The activity of SOD in the leaf sheath decreased in LJ11 while increased after 2 days of LTS and slightly decreased after 4 days of LTS in LJ25, consistent with the reports of Xiang et al., who reported that the antioxidant enzymes of leaves could increase under LTS within a certain period and then decline in different degrees^61^.

Previous studies have reported that the accumulation of ROS under LTS could trigger the expression of LT-related genes accompanied by physiological and biochemical changes^41,62^. *OSCOIN*, encoding a ring zinc finger protein, is known to be expressed in all the organs of rice and intensively induced by various abiotic stresses, such as LT, ABA, salt, and drought. The overexpression of *OsCOIN* could upregulate the expression of *OsP5CS*, enhance the proline content, and significantly improve the tolerance to LT, drought, and salt stresses^63^. In this study, the expression of *OsCOIN* in most investigate tissues were all upregulated under LTS on the reproductive stage of rice, consistent with previous reports^63^. This result indicated that *OsCOIN* played a positive role in response to LTS at the reproductive stage of rice in cold regions. *OsCATC*^47,48,64^, directly encoding catalase, whose expression under LTS was accompanied by the changes in CAT activity, was significantly upregulated in LJ25 while sharply downregulated in LJ11. *OsPOX1*, as a third peroxidase encoding gene, is known to be expressed in both aboveground and underground tissues and expressed in vessels and anthers when in the early flower stage of microspore development^37,65^. In this study, the expression trends of *OsPOX1* were similar to those of *OsCATC*, indicating that both *OsCATC* and *OsPOX1* could be used for enhancing the LT tolerance of rice in cold regions. Under LTS, ROS accumulation could also promote the signaling network response to LTS, such as the MAPKK-MAPK pathway^66^. *OsMAP1*, encoding a mitogen-activated protein, is involved in the MAPKK-MAPK pathway. Treatment for 48 h at 12 °C and the ROS accumulation during this time could positively^67^ induce *OsMAP1* expression; this is consistent with its expression pattern observed in LJ25 in this study, although not in LJ11. These findings indicate that *OsMAP1* plays a significant role in LT tolerance in rice in cold regions.

## Conclusions

**Figure 6:**
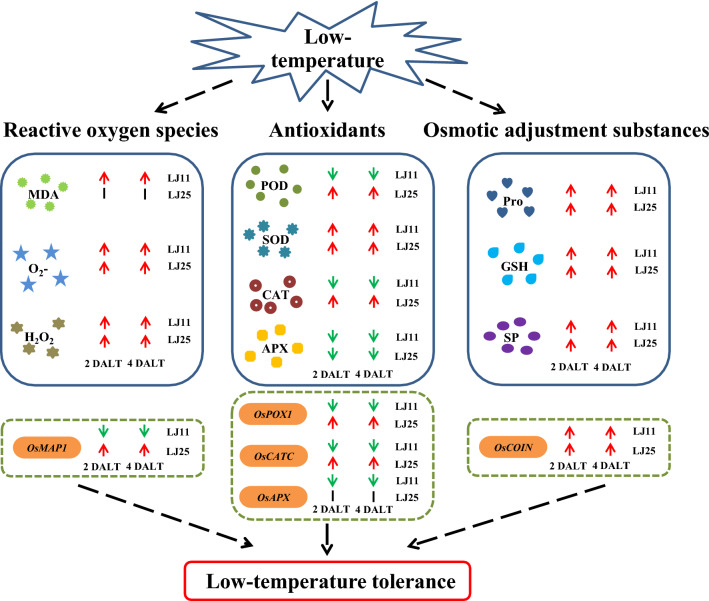
Model of the rice response to low temperatures in cold regions. Red arrows represent increased concentrations or expression levels. Green arrows represent decreased concentrations or expression levels, and black lines represent an absence of significant change in concentrations or expression levels.

In this study, by analyzing the responses of ROS, osmotic adjustment substances, and antioxidants on different tissues of rice under LTS at the reproductive stage, we derived at the following conclusions: First, the amounts of ROS and MDA were almost significantly increased in LJ11 while slightly increased or significantly decreased in LJ25 under LTS; the contents of those osmotic adjustment substances (Pro, SP, and GSH) were all significantly increased in both LJ11 and LJ25; the antioxidant enzymes, except the CAT and POD (upregulated in LJ25 and downregulated in LJ11), all others showed the same trends in LJ25 and LJ11. Second, most of the contents or activities in leaf sheath were less or weaker than those in flag leave and panicle. Third, the expression of the genes related to the antioxidant enzymes investigated here also showed the same trends with the phenotypic data of those enzymes. To sum up, our findings provided a conceptual model of the effects of LTS on the physiological and biochemical indices and gene expression patterns of rice in cold regions based on our data (Fig. 6) and laid a foundation for rice LT-tolerance breeding in cold regions.

## Supplementary Information

Supplementary Table S1.

Supplementary Table S2.

Supplementary Table S3.

Supplementary Table S4.

Supplementary Table S5.

Supplementary Table S6.

## Data Availability

The datasets generated during and/or analysed during the current study are available in Supplementary files.
